# Psychometric properties of the Jonsson-Abbott Scale: Rasch and confirmatory factor analyses

**DOI:** 10.3389/fpsyg.2022.936685

**Published:** 2022-09-14

**Authors:** David Forsström, Anders Kottorp, Alexander Rozental, Philip Lindner, Markus Jansson-Fröjmark, Per Carlbring

**Affiliations:** ^1^Department of Psychology, Stockholm University, Stockholm, Sweden; ^2^Department of Clinical Neuroscience, Center for Psychiatry Research, Karolinska Institutet and Stockholm Health Care Services, Stockholm, Sweden; ^3^Faculty of Health and Society, Malmö University, Malmö, Sweden; ^4^Department of Psychology, Uppsala University, Uppsala, Sweden; ^5^UCL Great Ormond Street Institute of Child Health, London, United Kingdom; ^6^Center for Dependency Disorders, Stockholm Health Care Services, Stockholm, Sweden

**Keywords:** Jonsson-Abbott Scale, gambling behavior, low-risk population, risks of gambling, psychometric analysis

## Abstract

Measuring and assessing the different aspects of gambling behavior and its consequences is crucial for planning prevention, treatment, and understanding the development of at-risk and problem gambling. Studies indicate that instruments measuring problem gambling produce different results based on the characteristics of the population assessed. To accurately measure at-risk and problem gambling behavior, especially in a low-risk population, measures must cover a wider set of dimensions than the negative consequences already manifest. The Jonsson-Abbott Scale (JAS) includes items that cover overconsumption, actions that reinforce gambling behavior, and belief in gambling fallacies, based on a three-factor structure and has previously demonstrated good psychometric properties. However, there is a need to investigate how the instrument also functions in low-risk populations. This study aims to do so using both confirmatory factor and Rasch analysis; this research included 1,413 Swedish participants who endorsed at least one JAS item. The results replicated the previous three-factor solution and indicated that the instrument had good reliability. In addition, the results demonstrated that the three factors are independent, and the overall score per factor needs to be analyzed. In summary, the JAS appears suitable for use in low-risk populations to measure various aspects of gambling behavior.

## Introduction

Excessive gambling often leads to negative consequences, primarily financial loss and harm (damaging and adverse consequences), both for the individuals who gamble and their families. Several ways of defining and categorizing harm from gambling have been suggested, including the distinction by Langham et al. (2016), which includes financial and negative mental health consequences such as anxiety and depression (Langham et al., 2016). The economic hardships and mental health consequences of gambling can have a crippling effect on the life of the individual that gambles. For example, Gray et al. (2021) found a robust relationship between gambling and suicide attempts in their scoping review. Gambling can also cause different types of criminal behavior (Binde et al., 2022). Moreover, excessive gambling affects those close to the gambler in a negative way, particularly children (Lorenz and Shuttlesworth, 1983; Lorenz and Yaffee, 1986; Riley et al., 2021; Irie and Kengo, 2022). Limiting harm is thus crucial for a population that engages in excessive gambling. Many studies on preventive efforts demonstrate only a small population-level impact on decreasing gambling behavior (McMahon et al., 2019; Forsström et al., 2020b). However, screening high-spending customers seems to be a promising preventive endeavor (Jonsson et al., 2019, 2020). An important aspect of limiting harm is developing ways to screen different populations to determine the risk of gambling-related problems and understand the trajectories of harm caused by gambling.

Determining where an individual is located on a harm continuum and determining the level of negative consequences is vital for analyzing the steps necessary for harm reduction. Several instruments have been developed to assess gambling problems using similar and sometimes overlapping constructs from different perspectives. These include the South Oaks Gambling Screen (Lesieur and Blume, 1987), the NORC Diagnostic Screen for Gambling Problems (Gerstein et al., 1999), and the Problem Gambling Severity Index (PGSI) (Ferris and Wynne, 2001; Wynne, 2003). Importantly, some studies have indicated that instruments measuring the risk of developing gambling problems and already manifesting gambling problems may behave differently in a low gambling population. Different factor structures have been found in low gambling populations (Holtgraves, 2009; Forsström et al., 2020a). Items used in low gambling populations also seem to function in terms of gender, with men and women differing in endorsing items (Forsström et al., 2021). This implies that the consequences of gambling and the amount of time and money spent might be less relevant when trying to appraise the risk among individuals with a low level of gambling, which may still be problematic and/or entail a future risk. At the same time, simply exploring attitudes and motives of gambling might not be enough to assess risk in the target population. Furthermore, exploring other aspects than time and money spent and negative consequences of gambling, such as attitudes and reasons for gambling, is important, as gamblers underestimate their losses (Auer and Griffiths, 2017; Heirene et al., 2021). Also, one study found that individuals who did not complete an online screener for gambling because they wanted to avoid psychological distress (Peter et al., 2021). Therefore, using other types of items covering attitudes and/or other behaviors might be relevant.

To understand the risks among low-gambling populations, we developed the Jonsson-Abbott Scale (JAS) (Jonsson et al., 2017a), covering three pertinent aspects of gambling: overconsumption, reinforcement of gambling behavior, and gambling-related fallacies. JAS was developed as part of the Swedish prevalence study, the Swedish Longitudinal Gambling Study (Swelogs) (Romild et al., 2014). The goal was to create an instrument that could be used to identify early signs of future gambling problems and also to investigate the relationships between the indicators chosen.

Three constructs were created: Reinforcers, Overconsumption and Gambling fallacies. Items belonging to the Reinforcers. Items in the first factor were selected to cover both the positive and negative reinforcement of gambling (Ramnerö et al., 2019). The Overconsumption factor covers different aspects of gambling more than intended and difficulties in trying to abstain from gambling. The Gambling fallacies factor covers items on misconceptions about gambling, such as believing that you could make money in the long run from gambling and that winning money is related to an individual's skill level. The number of items included in the instrument was not based on an iterative process where items were selected from a larger pool of items; instead, the extent of the scale was upper bound by the number of items that could be included in the Swelogs questionnaire. The items are scored from one to seven using a Likert-scale, with one meaning “Do not agree at all” and seven meaning “Agree completely”.

Thus far, only one previous study by Jonsson et al. (2017a) has examined the psychometric properties of the JAS. The proposed three-factor solution had an acceptable fit, the reliability of the instrument was at a satisfactory level, and the three factors were significant predictors of risk potential (Jonsson et al., 2017a). However, there is an urgent need to investigate the psychometric properties of JAS further for several reasons. First, essential aspects of sample characteristics were not reported in Jonsson et al. (2017a), making it difficult to assess the applicability of findings across different populations. Second, the CFA results were inconclusive, which warrants further testing in other samples. In the present study, Rasch analysis was used, for the first time, to answer research questions regarding response functions.

The purpose of the current study was to continue examining the psychometric properties of the JAS by examining psychometric aspects not addressed in past research, including using a well-characterized, low-risk sample using modern psychometric approaches grounded in the ontological-causal measurement framework of Borsboom et al. (2004), wherein validity rests on the assumptions of the construct measured existing and having a causal impact on the responses (here, presumably mediated through insight). This study takes a pragmatic stance when exploring the psychometric properties of the scale. The research questions were chosen on the assumption that the results would be applicable in a discussion about the practical use of the scale. Thus, mixing analytic techniques from classical test theory with applications from item response theory (e.g., Rasch analysis).

The following research questions are addressed:

### Evaluation of the validity of the instrument

Is the three-factor solution viable in a low-risk population?

What are the correlations with other gambling instruments?

How do the different factors relate to each other based on the results of the person-item map?

How were the INFIT values for the included items?

Do the items function differently between men and women and with age?

Do respondents endorse the seven scale steps?

How did the rating scale function for the instrument?

### Precision of the scale

What are the reliability coefficients on a whole scale and at a factor level?

What are the person separation index and item separation index for the instrument?

## Methods

### Procedure

A survey (available only in Swedish) was created by the authors (available at https://osf.io/s287j/) as part of a larger project to collect data for the psychometric evaluation of gambling-related instruments. The survey included several instruments, of which the following were used in the current study: the GamTest (Jonsson et al., 2017b; Forsström et al., 2020a, 2021) and Problem Gambling Severity Index (PGSI) (Ferris and Wynne, 2001) from the Swedish Longitudinal Gambling Study (SWELOGS) (Romild et al., 2014). In total, 85 questions were used from SWELOGS, all of which were multiple choices, including the JAS (Jonsson et al., 2017a). The questions covered the types of gambling activity, its frequency, and the amount of time and money spent. To avoid order effects, all the included instruments were presented randomly to the respondents.

The participants were recruited *via* a state-procured online survey company. The eligibility requirements for the survey were as follows: (1) age between 18 and 85, (2) fluency in Swedish, and (3) access to a computer. Information on the study and a link to the survey were sent *via* email. A total of 5,000 potential respondents, selected to match the general Swedish population in terms of age and sex, received an e-mail with the option to answer the survey. Three reminders were sent out after the initial mail inviting them to participate. No compensation was provided for participation. Every question was set as compulsory, with no missing data from those that completed the survey. All respondents consented to participate in the study before answering the survey questions. A total of 2,257 participants completed the entire survey out of a total of 2,376, indicating a 95% completion rate and a combined opt-out and dropout rate of 47.5 % relative to the 5,000 initially invited to participate. Of the completers, 23 respondents took longer than 2 days to complete the survey and hence were deemed indicative of poor response quality and thus omitted. In summary, data from 2,234 respondents were eligible for analysis. The group that started but did not complete the survey had a lower mean age and lower mean income than the respondents that completed the entire survey. The age difference was 4 years, and the difference in income was ~5.500 Swedish kronor. More information on the 121 respondents that started but did not complete the survey is available in Forsström et al. (2020b).

### Participants

In total, 2,234 respondents completed the survey, with a gender distribution of 53% men (*n* = 1,184), 47% women (*n* = 1,048), and two identified as “other” (0.1%). The average age was 51.4 years (SD = 16.2), with men [52.4 years (SD = 16.2) years] being slightly older than women [50.3 years (SD = 15.5) years].

As many validity aspects would suffer from floor effects, the psychometric analyses were conducted on the subsample (*n* = 1,413) that endorsed at least one item of the scale. Hence, all included respondents scored at least 12 points, one point higher than the theoretical minimum score of 11. None of the 1,413 that scored 12 or over were excluded from the study. A score of 11 means that a respondent has answered “do not agree at all” on all the items meaning that what was described in the items had not been experienced by the respondent. This smaller sample contained 850 men (60.2%) and 563 women (39.8%). The mean age of the smaller sample was 50.5 years (SD = 16.1), with men and women being 51.3 (SD = 16.6) and 49.4 (SD = 15.2) years, respectively. There was no significant difference in mean age between men and women in the sample, *t*_(1, 411)_ = 2.14, *p* = 0.033.

**Figure 1:**
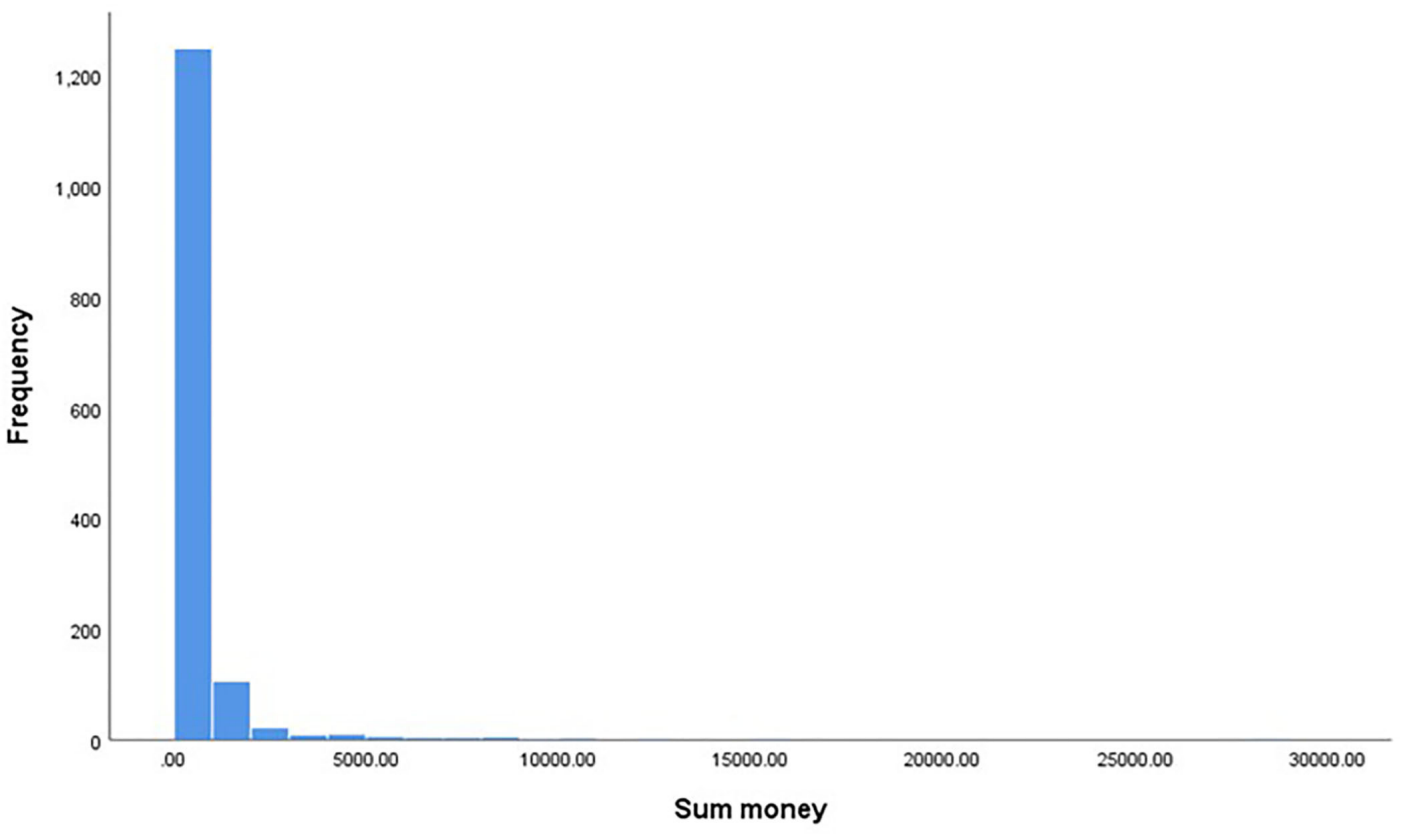
Frequency distribution of total bet amount.

For the subsample, the amount of money spent on gambling was a composite measure based on the spending of individual types of gambling during the last 30 days, which was divided to represent spending per week (eight different types of gambling activities were included, e.g., casino gambling, poker, and betting). The mean amount spent on gambling was 514 Swedish kronor (SD = 1.345) per month (~$55). The distribution of spending is depicted in Figure 1. Spending ~125 Swedish kronor per week. There is no consensus in the extant literature on what constitutes low-risk gambling; one influential study revealed, however, that gambling expenditure under 75 Canadian dollars per month (~ 544 Swedish Kronor) was associated with low-risk gambling (Currie et al., 2017). The study by Currie et al. (2017) and our study contain a similar population regarding mean age and gender distribution. Against this reference, the recruitment strategy successfully gathered data from a low-risk sample, congruent with the study aims.

For the 821 respondents who did not endorse any of the JAS items, the gender distribution was 40.7% for men, 59.1% for women, and 0.2% for “Other”. The mean age for the entire group was 52.8 (SD = 15.24); for men, it was 55.3 (SD = 16.51), and for women, 51.2 (SD = 15.78). There was a significant difference in mean age between the included 1,413 and the excluded 821, *t*_(2, 232)_ = 3.24, *p* < 0.001.

Of the 821 respondents, 539 did spend any money on gambling. The mean spending on gambling was 61 Swedish kronor per month compared to the included 1,413, which spent 512 Swedish kronor per week. There was a significant difference in spending on gambling, *t*_(2, 232)_ = 9.58, *p* < 0.001.

The maximum amount anyone spent in the group of 821 respondents was 1,735 kronor per month. Of the 821 respondents, 813 had a total score of zero on the PGSI, six participants had a score of one on the PGSI, and two respondents had a score of seven.

In all, the excluded 821 differed regarding gender distribution, mean age, and spending on gambling.

### Statistical analyses

To allow comparison with past research and examine the novel research questions, both analyses from classical test theory and polytomous Rasch analysis were used. For all analyses based on classical test theory and correlations, Jamovi version 0.9.2.9, SPSS V.25, or R v.3.6.1 was used (IBM Corp. Released., 2017; The Jamovi Project, 2021). Weighted least squares mean and variance-adjusted confirmatory factor analysis (CFA) were used for the latent structure of the JAS (nlminb optimization method), given that the data were skewed. Such an approach has been demonstrated to perform better in the likelihood that sample scores have a skewed distribution (Beauducel and Herzberg, 2006). To assess the fit of the three-factor structure, we used different cutoffs. The χ^2^-values should be less than the degrees of freedom (Sun, 2005), and the model should be non-significant. Hu and Bentler (1998) recommends an RMSEA over 0.06 for samples larger than 250 respondents (our sample contains 1,413 respondents). The comparative fit and Tucker–Lewis index should ideally exceed 0.90 (Hu and Bentler, 1998). The standardized root-mean-square residual should be <0.08 to indicate a good fit (Hu and Bentler, 1998). Omega was used to assess the general internal reliability estimate for the 11 items. Omega has several advantages over Cronbach's α, such as more realistic assumptions, leading to less inflation and attenuation issues regarding internal consistency (Dunn et al., 2014). The recommendations provided by Dunn et al. (2014) were employed to calculate omega values (Cronbach's alpha is also included for comparison). *T*-tests were used to explore differences in mean age and spending on gambling. A Bonferroni correction (Perrett et al., 2006) was carried out to limit the familywise error. The significance was set to *p* = 0.017 based on the significance level of 0.05 divided by the number of tests, which was three. Finally, traditional convergent validity was assessed by calculating correlations with PGSI and GamTest scores.

A polytomous Rasch analysis was implemented to assess response functioning using WINSTEPS software (3.9.1.0.0), according to the steps described by Lerdal et al. (2016). Rasch analysis involves the transformation of raw scores from the JAS into equal-interval measures for items and persons by using a logarithmic transformation of the odds probabilities (Bond and Fox, 2013). In this study, two additions were made to the analysis proposed by Lerdal et al. (2016). First, an initial log-likelihood χ2-analysis was used to explore if the data were suitable for a rating scale model (RSM) or a partial credit model (PCM), with each item having its own rating scale structure. Second, an initial investigation of standardized residual correlations between the included items in the JAS was performed, given the assumption of local independence in Rasch models (Fischer, 1995). A criterion of r > 0.5 (shared variance of > 25% variance) was set as a sign of dependency of pairs of items to determine whether the data were suitable for Rasch analysis. When examining the standardized residual correlations between the JAS items, none of the pairs exceeded the criterion of *r* > 0.5. The correlations ranged from −0.24 to 0.25. Hence, the data used supported the assumption of local independence, allowing us to proceed with our analysis.

After the two abovementioned analyses confirmed that the data were suitable for conducting a Rasch analysis, the JAS rating scale categories were applied according to the following criteria: (1) a minimum of 10 responses per step category, (2) the average measures should advance monotonically for each step category, and (3) values <2.0 on the outfit mean square for the step category calibrations (i.e., values close to 1.0 suggest fit, and values over 1.0 indicate possible deviations from the model) (Linacre, 2002). When the criteria were not met, the next step was to collapse the rating scale categories or delete categories, as suggested in the literature (Linacre, 2004). The internal structure of the measure was examined by goodness-of-fit statistics; that is, mean square residuals and standardized z-values, which indicate a match between actual responses to the items and the expected responses, in line with Rasch model assertions (Bond and Fox, 2013). Infit statistics were used to assert goodness-of-fit for items. Infit statistics are considered more sensitive than outfit statistics regarding item performance and more informative when determining internal scale validity (Wright and Masters, 1982; Bond and Fox, 2013). The mean square fit statistic is often preferred when examining the goodness-of-fit of items with polytomous data, as it is not as affected by sample size (Smith et al., 2008). The item goodness-of-fit for the infit mean square was set between 0.7 and 1.3 (Smith et al., 2008). If one or more items did not exhibit acceptable goodness-of-fit according to the model, the items were considered for removal from the analysis. The iteration process was repeated until all items met the criterion of 0.7–1.3. To examine the level of precision of the converted measures, item separation indices were calculated (Fisher, 1992). By investigating the range and precision of the person and item estimates, the person separation index reflects the number of statistically different groups that the measure can identify in a specific sample. This was used to see if the participants could be separated into groups and if these groups indicated increased risk and endorsement of items in the subscales going from endorsing items in Reinforces to Overconsumption and, as a final step, items in Gambling fallacies. The goal was to explore the increase in risk and not the presence of risk. The item-separation index works similarly, reflecting the number of statistically different groups that a given sample can identify among the items. Here, an index >1.5 ensures that the JAS can differentiate between a minimum of two different groups when examining both the sample and the items (Wright, 1996). Finally, differential item functioning (DIF) analysis was applied to explore whether the response patterns of the JAS were stable across both age and gender. To determine the magnitude of DIF, the Mantel-Haenszel statistic for polytomous scales using log-odds estimators was implemented (Mantel, 1963), with a criterion set at *p* < 0.01 to adjust for mass significance. For age, the sample was split into two parts: young and old. The birth year median was used for classification, resulting in 714 participants up to 1963 and 699 participants from 1964. This was done to have two separate comparison groups of equal size. Dividing the sample into more groups would have resulted in small groups, making it hard to compare the different age groups. The mean age for the older group was ~64 years (SD = 8.0), and for the younger group, 36.8 years (SD = 9.2).

### Ethical considerations

The study was approved by the Swedish Ethical Review Authority (Dnr: 2014/545 and 2020-02923). Before responding to the survey, all respondents checked a box confirming that they had read and understood all information regarding the study, thereby providing their informed consent. Participation was voluntary, and the respondents could end their participation whenever they wanted. No compensation was provided. The study information included full disclosure of the nature of the study, and the principal investigator's email address and telephone number were provided. The respondents were also informed that they could request a summary of the results by contacting the principal investigator but that individual data could not be obtained.

## Results

### Results from the analysis that investigate the validity

The results of the CFA are presented based on the recommendations of Jackson et al. (2009) and Cabrera-Nguyen (2010). For the subsample of gamblers who scored over 11 points on the JAS, the CFA was used to test the three-factor solution suggested by Jonsson et al. (2017a). The result of the $\chi_{\left(41\right)}^{2}$ = 40.793, *p* = 0.480. Both of these prerequisites were fulfilled. The root-mean-square error of approximation (RMSEA) was 0.068 with a 95% confidence interval of 0.049–0.088. The comparative fit index was 0.914, and the Tucker-Lewis index was 0.885. As stated, these values should exceed 0.90 (Hu and Bentler, 1998). Thus, the goodness-of-fit values were over or close to the recommended values. The standardized root-mean-square residual was 0.045; a value <0.08 indicates a good fit (Hu and Bentler, 1998). The results of the CFA are presented in Table 1. When examining the values from the factor analysis in relation to the cutoffs, the three-factor structure is valid, has an acceptable fit for the sample, and is better than in Jonsson et al. (2017a).

**Table 1:** Factor loadings of the Jonsson-Abbott Scale.

**Factor**	**Indicator**	**Estimate**	**SE**	**Z**	**p**	**Standardized estimates**
Reinforcers	I gamble for the excitement	1.000^a, b^				0.431
	Gambling is among the most enjoyable things there are	1.354	0.063	21.588	<0.001	0.772
	Gambling can make me forget everything else for a while	1.098	0.078	14.009	<0.001	0.671
	My gambling gives me friends	0.771	0.086	8.967	<0.001	0.615
Overconsumption	I gamble for more money than intended	1.000^a^				0.842
	I gamble a longer time than intended	0.957	0.035	27.073	<0.001	0.886
	I gamble when I should have done other things	0.796	0.046	17.278	<0.001	0.734
	When gambling, I find it hard to stop	0.884	0.041	21.386	<0.001	0.780
Gambling fallacies	My gambling is a way to make money	1.000^a^				0.598
	When I win, it is due to my skill	1.294	0.110	11.800	<0.001	0.691
	If I just gamble enough, my gambling will pay off	1.032	0.060	17.088	<0.001	0.804

a Fixed parameter.

b For all of the questions, scale-steps ranged from “Do not agree at all” (1) to “Completely agree” (7).

The correlations between the JAS, PGSI, and GamTest were relatively high and significant, as presented in Table 2.

**Table 2:** Correlations.

	**JAS**	**Reinforcers**	**Gambling fallacies**	**Overconsumption**	**PGSI**	**Gam test**
JAS						
Reinforcers	0.877^**^					
Gambling fallacies	0.857^**^	0.590^**^				
Overconsumption	0.813^**^	0.593^**^	0.572^**^			
PGSI	0.488^**^	0.319^**^	0.378^**^	0.550^**^		
GamTest	0.649^**^	0.458^**^	0.487^**^	0.708^**^	0.827^**^	

** *p* < 0.01.

**Figure 2:**
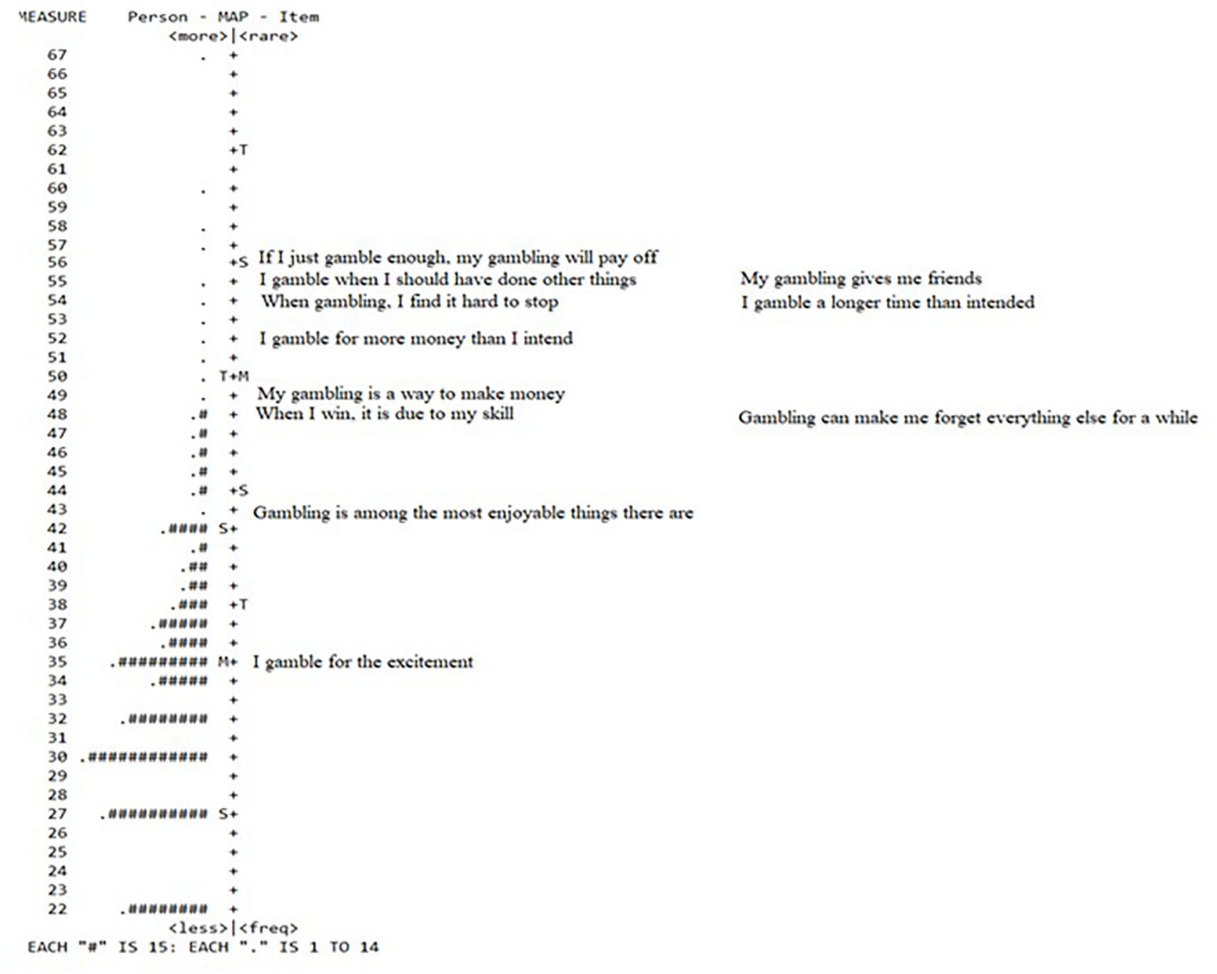
Person-item map JAS (11 items).

Most respondents were at the bottom end of the spectrum, indicating that they did not endorse high scores on several items and/or endorsed several items. The endorsed items follow an interesting pattern in that the items that are endorsed to the highest degree are three items from the factor reinforcers (“I gamble for the excitement,” “Gambling is among the most enjoyable things there are,” and “Gambling can make me forget everything else for a while”). Two gambling fallacy items (“When I win, it is due to my skill” and “My gambling is a way to make money”) follow after the three reinforcer items. The four overconsumption items follow thereafter. The least endorsed items are: “My gambling gives me friends” (reinforcers) and “If I just gamble enough, my gambling will pay off' (gambling fallacy). See Figure 2 for details on endorsing the items in the JAS.

The item goodness-of-fit was carried out based on the factor solution proposed in Jonsson et al. (2017a) on the three-factor solution. Two items in the subscale reinforcers demonstrated a misfit: “My gambling gives me friends” (infit value of 2.05) and “Gambling can make me forget everything else for a while” (infit value of 1.36). The item gambling fallacy did not contain any misfit items. The item overconsumption had one item that demonstrated a misfit: “I gamble when I should have done other things” (with an infit value of 1.4).

The results of the rating scale analysis demonstrated that scale steps six and seven were reversed, with more respondents endorsing scale step seven than six, indicating a violation of the predefined set criteria. In a subsequent analysis, once scale steps six and seven were collapsed, the scale step fulfilled the predefined criteria. The results indicate a change in response to alternatives to the questions.

### Practical use of the scale

On an overall scale, the reliability was *r* = 0.892 (for all items measured, Cronbach's alpha was 0.873). All factors had a high Omega value, ranging from 0.713 to 0.882. Reinforcers, overconsumption, and gambling fallacy had an Omega of 0.713, 0.882, and 0.750, respectively. The Cronbach's alpha for the subscales was 0.687 for the subscale Reinforcers, 0.885 for the subscale Overconsumption and 0.721 for the subscale gambling fallacy.

A total of 1,413 respondents endorsed one or several JAS items. The results presented in Table 3 summarize these responses.

**Table 3:** The psychometric properties of Jonsson Abbott scale.

	**JAS total scale (11 items)**
	**(*N* = 1,413)**
Rating scale functioning	All criteria met
Person misfit	
*N* (%)	1,032 (73%)
Maximum score	77
Minimum score	12
Person separation index	
Reinforcers	0.97
Gambling fallacy	0.25
Oversconsumption	0.70
Item separation index	
Reinforcers	27
Gambling fallacy	11.07
Overconsumption	2.92
Differential Item Functioning (DIF)	No difference for sex and age

For the subscale reinforcers, the person separation index for the two items was 0.97, with a reliability of 0.49. The person separation index was 0.25, with a reliability coefficient of 0.60 for the subscale Gambling fallacy. Overconsumption had a separation index of 0.70 and a reliability of 0.33. This indicates that, on a factor level, different groups of respondents cannot be differentiated based on their scores.

The factor reinforcers had an item separation index of 27 with a reliability of 1.00, and gambling fallacies had an item separation index of 11.07, with a reliability of 0.99. Overconsumption had an item separation index value of 2.92 with a reliability of 0.90. All the values obtained at the factor level indicate that the sample was large enough to confirm the item difficulty hierarchy (Souza et al., 2017).

The results demonstrated no significant differences in item functioning. Age was divided into two groups (young and old), and no significant differences were noted. Furthermore, there were no significant differences in gender. However, one item from reinforcers, “Gambling is among the most enjoyable things there are,” had *p* = 0.0146, with men endorsing the item more. However, this difference did not survive correction for multiple comparisons.

## Discussion

### Model fit and validity

Based on the CFA results, the three-factor solution proposed for the JAS had an overall good fit. Two of the fit measures were close to the fit indices proposed by Hu and Bentler (1998). However, these two measures were close to the recommended scores, and the confidence interval for RMSEA overlapped with the suggested benchmark for a good fit. It is possible to conclude that the three-factor structure suggested by Jonsson et al. (2017a) is viable for use in a population characterized by low risk. In line with the framework for JAS, the results of the study suggest that individual factors should be used as indicators of at-risk gambling, as using the total score might not reflect the development of risks associated with gambling. Additionally, the person-item separation map indicates that the items are endorsed on an overall factor-by-factor level (see Figure 1).

The JAS had a fairly high correlation with both the PGSI (Ferris and Wynne, 2001) and the GamTest (Forsström et al., 2020a, 2021), indicating that it partly addresses a problem gambling construct and can measure the incidence of at-risk and problem gambling. Also, JAS had a higher correlation with GamTest, which contained more items and can also be seen to address the risk progression. Furthermore, the overconsumption subscale was highly correlated with both GamTest (Forsström et al., 2020a, 2021) and PGSI (Ferris and Wynne, 2001). Further, the other factors can be regarded as markers of the onset of at-risk and problem gambling, as the endorsement of the items occurs in three steps.

As previously mentioned, instruments that examine levels of risk or gambling problems may perform poorly in low gambling/low-risk populations in terms of factor structure and different aspects of model fit (Holtgraves, 2009; Forsström et al., 2020a, 2009). However, the results of the current study suggest that the JAS might be suitable in a low-risk population and that factor structure and model fit are similar to those used in a high-gambling population. Future studies are needed to explore the function of gambling instruments in different populations.

Based on the item goodness-of-fit, several items can be deleted from the three factors—reinforcers, gambling fallacies, and overconsumption. The only item with a clear misfit and a high value was 'My gambling will give me friends'. The other two items with a misfit were close to the suggested cut-off values (Smith et al., 2008); therefore, deleting these two items was unnecessary. However, one suggestion is to delete the item ‘My gambling will give me friends' since it was least likely to be endorsed and, thus, does not add much to the degree of validity of the instrument. There is, however, a risk that when deleting an item demonstrating misfit, other items show up as new misfits (REF). If successful, such processes can shorten the instrument, minimize the burden for the respondents, and create a more unidimensional measure of the target phenomenon. This should therefore be explored with the JAS scale in the future.

Another reason for keeping the two items is to maintain the content validity of the instrument. Both items are indicators of increased involvement in gambling and are precursors of gambling problems. In populations with higher levels of risk, these items are pertinent to detecting risk.

Based on the results, it is possible to argue that JAS has a high degree of validity in relation to the paradigm that Borsboom et al. (2004) suggest is based on describing the basis of the assumptions of the instrument.

### Reliability of the Jonsson-Abbott scale

The results of both scale and factor levels are mixed; on the one hand, the findings suggest that the JAS has good reliability based on the benchmarks indicated by Streiner (2003). Higher reliability could indicate redundant items (Streiner, 2003). The reliability of the sample used in this study was higher than that reported by Jonsson et al. (2017a). A plausible reason could be that because the sample used in our study endorsed fewer items, a higher reliability coefficient across factors was created. On the other hand, the low separation indices indicate that each factor is not sensitive enough to detect even two distinct groups in the low-risk sample. Hence, we do not have a distinct measure of the target phenomenon, as all people end up in the same group. This can be a consequence both in relation to a low-risk sample but also in the construction of the instrument. A better targeted JAS instrument with more items that are better matched to the low-risk sample could be a possible solution to explore and evaluate. Combining items from factors could be a logical first step to explore. However, this approach then needs to meet the criteria for unidimensionality as well to improve the precision of the target construct.

### Practical implications for the JAS

The results from the person item map indicate a hierarchy regarding the subscales. From a theoretical perspective and in line with Jonsson et al. (2017a), it appears that behavior that reinforces gambling is the first indicator of possible gambling problems, as it is most common in a low-risk population. The items most likely to be endorsed belonged to the factor Reinforcers, followed by two items in the factor Gambling fallacies. Four items belonging to overconsumption followed. The least endorsed items, 'My gambling will give me friends' and 'If I just gamble enough, my gambling will pay off', can be understood as red flags in the assessment of gambling problems in the sample. The item 'My gambling will give me friends' also demonstrates a misfit. One suggestion, as mentioned earlier, could be to delete this item from the scale and replace it with a new item measuring Reinforcers. These findings converge with the results of the three-factor structure, indicating that the scale consists of separate factors that should not be combined into a total score.

This study results also imply that the scale should be used on a factor—not the whole-scale level, based on the person-item map results. Additionally, the JAS instrument was not designed to analyse only one construct but several aspects of gambling. On the other hand, the low separation indices at the factor level are an argument against this solution, at least in the current form of JAS. This result might be an example of an overall low endorsement of the items by this population. If the JAS is used in different and more high-risk samples in future studies, it may be able to further differentiate between levels of gambling severity.

When administering the JAS in an online setting, a three-step scale consisting of reinforcers, gambling fallacies, and overconsumption is presented. If an individual does not endorse the item of time belonging to the factor reinforcers, there is a low probability, according to the person-item map, that the individual will endorse the items in the overconsumption factor. Therefore, the scale could be administered in an item-response manner using the person-item map as a blueprint. This is further substantiated by the predictive validity for at-risk and problem gambling pertaining to the Reinforcers and Gambling fallacy (Jonsson et al., 2017a), which indicates the value of initial screening using items from the above two factors.

There were no differences in how the factor items functioned in relation to age and gender in the included sample. This result suggests that the JAS can be used across populations, and no special considerations are required when using the scale.

However, of the 821 respondents that did not endorse any item on the scale, 282 of them spent money on gambling. This, along with the non-gamblers, can cause floor-constrained data, affecting the outcome of the analysis (especially the CFA), and these responders were excluded. The interesting question is why these gamblers did not endorse any items. One reason could be their low-level gambling. Furthermore, around 58% of the 282 were women, which might indicate that the questions might have a bias toward men and that the items in the subscale Reinforcers that should be endorsed are based on typical male reinforcers. There is a need to analyse the demographics and spending of non-responders to further understand the use of the instrument.

### Limitations

Although the present study reveals important findings, it has several limitations. Approximately half of the invited respondents answered the survey, which increases the chances of a self-reported bias in the sample. The consequence of self-report bias has been demonstrated in previous studies (Griffiths, 1994; McCusker and Gettings, 1997). Furthermore, only 1,413 of the 2,234 participants endorsed one or more items on the scale. Therefore, the means reported in the current study should not be interpreted as representing the average population.

### Future research

The findings from the current study suggest that future research should focus on examining how the validity of JAS functions when gamblers are on a continuum, ranging from lower to higher-risk populations, and examine the endorsements of items by gamblers on the continuum of at-risk and problem gambling. Also, interviewing gamblers ranging from recreational to problem gamblers can help understand the common perception of the JAS.

In particular, the results indicate that the JAS instrument provides the most accurate description of the items at the factor level. In a study regarding PGSI, it was reported that some respondents did not grasp all the items in the PGSI (Samuelsson et al., 2019), which in turn may have adverse implications for the administration and scoring of the PGSI. The same may be true for JAS and should, therefore, be examined further in future studies.

### Conclusion

JAS incorporates three factors that describe the progression of at-risk and/or gambling problems. From that perspective, our study has demonstrated that the instrument has a high degree of validity in relation to how the measure was intended to function for a low-risk population. The results of the analyses are in line with how the instrument is conceptualized. However, individuals that do not endorse items but have low-level gambling should be explored when using the scale.

## Data availability statement

The raw data supporting the conclusions of this article will be made available by the authors, without undue reservation.

## Ethics statement

The studies involving human participants were reviewed and approved by the Swedish Ethical Review Authority (Dnr: 2014/545 and 2020-02923). The patients/participants provided their written informed consent to participate in this study.

## Author contributions

DF: conceptualization, methodology, validation, formal analysis, investigation, data curation, writing—original draft, writing—review and editing, supervision, and project administration. AK and PL: validation, formal analysis, and writing—review and editing. AR: validation, investigation, writing—review and editing, and project administration. MJ-F: conceptualization, methodology, validation, writing—review and editing, and supervision. PC: conceptualization, methodology, validation, writing—review and editing, supervision, and funding acquisition. All authors contributed to the article and approved the submitted version.

## Funding

The data collection was funded by the independent Svenska Spel's research council.

## Conflict of interest

DF is the Chief Behavioral Scientist at VakandiData. PL reports several past, ongoing, and planned industry-academia collaborations, yet has never received any direct reimbursement from the gambling industry, nor owns any stake therein. The remaining authors declare that the research was conducted in the absence of any commercial or financial relationships that could be construed as a potential conflict of interest.

## Publisher's note

All claims expressed in this article are solely those of the authors and do not necessarily represent those of their affiliated organizations, or those of the publisher, the editors and the reviewers. Any product that may be evaluated in this article, or claim that may be made by its manufacturer, is not guaranteed or endorsed by the publisher.
